# Oxidative stress improves coronary endothelial function through activation of the pro-survival kinase AMPK

**DOI:** 10.18632/aging.100569

**Published:** 2013-06-23

**Authors:** Ehtesham Shafique, Wing C. Choy, Yuhong Liu, Jun Feng, Brenda Cordeiro, Arthur Lyra, Mohammed Arafah, Abdulmounem Yassin-Kassab, Arthus V.D. Zanetti, Richard T. Clements, Cesario Bianchi, Laura E. Benjamin, Frank W. Sellke, Ruhul Abid

**Affiliations:** ^1^ Cardiovascular Research Center, Division of Cardiothoracic Surgery, Department of Surgery, Rhode Island Hospital, Providence, RI 02903, USA; ^2^ Warren Alpert Medical School of Brown University, Providence, RI 02903, USA; ^3^ Alfaisal University, College of Medicine, Riyadh 11533, KSA; ^4^ Faculdade de Ciencias Medicas da Santa Casa de Sao Paulo, Brazil; ^5^ Imclone, New York, NY 10016, USA

**Keywords:** Endothelial function, signal transduction, NADPH oxidase, reactive oxygen species, autophagy, aging

## Abstract

Age-associated decline in cardiovascular function is believed to occur from the deleterious effects of reactive oxygen species (ROS). However, failure of recent clinical trials using antioxidants in patients with cardiovascular disease, and the recent findings showing paradoxical role for NADPH oxidase-derived ROS in endothelial function challenge this long-held notion against ROS. Here, we examine the effects of endothelium-specific conditional increase in ROS on coronary endothelial function. We have generated a novel binary (Tet-ON/OFF) conditional transgenic mouse (Tet-Nox2:VE-Cad-tTA) that induces endothelial cell (EC)-specific overexpression of Nox2/gp91 (NADPH oxidase) and 1.8±0.42-fold increase in EC-ROS upon tetracycline withdrawal (Tet-OFF). We examined ROS effects on EC signaling and function. First, we demonstrate that endothelium-dependent coronary vasodilation was significantly improved in Tet-OFF Nox2 compared to Tet-ON (control) littermates. Using EC isolated from mouse heart, we show that endogenous ROS increased eNOS activation and nitric oxide (NO) synthesis through activation of the survival kinase AMPK. Coronary vasodilation in Tet-OFF Nox2 animals was CaMKKβ-AMPK-dependent. Finally, we demonstrate that AMPK activation induced autophagy and thus, protected ECs from oxidant-induced cell death. Together, these findings suggest that increased ROS levels, often associated with cardiovascular conditions in advanced age, play a protective role in endothelial homeostasis by inducing AMPK-eNOS axis.

## INTRODUCTION

Increased levels of reactive oxygen species (ROS) are often associated with microvascular pathology in age-related cardiovascular diseases (CVD) including coronary artery disease (CAD) and ischemic heart disease (IHD) - the major causes of death and morbidity in the USA[1-3]. These observations have led to the current paradigms in the field of cardiovascular and stroke research that reduction in ROS levels in the vessel walls should improve vascular functions [4]. However, interventional clinical trials using global antioxidants, e.g. HOPE, ATBC [5-8], have largely produced negative results in reducing primary endpoints of cardiovascular death and morbidity (with some suggestion of potential harm). Recent studies by us and others using genetically manipulated animal models and/or antioxidants in cultured endothelial cells (ECs) demonstrated that reduction in ROS did not improve vascular functions[9-12]. Instead, reduced ROS levels resulted in the disruption of the signal transduction events that are essential for nitric oxide (NO) generation in vascular endothelium[10, 13-15], which in turn impaired vasodilatation[10, 14, 16]. Together, these findings suggest that increased ROS levels often present in elderly patients with CVD may play critical roles in endothelial function.

Precise mechanisms by which ROS modulate signal transduction in ECs are not fully understood. Several studies have shown that ROS play critical roles in ECs by activating signaling intermediates including PI3K-Akt-eNOS, PLCγ1, PKC and ERK1/2 [9, 17, 18]. Recent reports suggest that the pro-survival kinase AMPK, which becomes activated during cellular stress including caloric restriction and reduction in AMP/ATP ratio, can also be regulated by oxidants generated during hypoxic or fluid shear stress in ECs [19-21]. AMPK, in turn, is known to regulate a number of signaling intermediates and transcription factors including HIF-1α, FOXO1 and PGC-1α [22, 23], resulting in increased EC survival and proliferation. One of the major protective roles of AMPK in cell survival is carried out through its regulatory role in autophagy [21, 24], a cellular process for the recycling and re-utilization of the damaged macromolecules and organelles using intracellular lysosomal degradative pathway [24, 25]. However, it is not known whether ROS regulate activity of the survival kinase AMPK and autophagy in ECs.

There are several sources for intracellular ROS in EC including NADPH oxidases, mitochondria, cytochrome P450 and xanthine oxidase. Rac1-dependent NADPH oxidase is a major source of endothelial ROS, which is a multi-subunit enzyme complex containing membrane-bound gp91phox (Nox2) and p22phox subunits, and cytosolic p47phox, p67phox and Rac1[12, 26, 27]. Several other NADPH oxidases are present in ECs, e.g. nox4, nox1, nox5 [11, 28-31]. Nox2 containing NADPH oxidase is present in different subcellular compartments in ECs including cell and perinuclear membrane, and endoplasmic reticulum (ER)[27, 32]. Recent work from others and our labs has shown a previously unappreciated role for NADPH oxidase-derived ROS in the activation of downstream eNOS to synthesize NO[9-11, 14, 15, 33, 34]. These studies suggest that NADPH oxidase-derived ROS play a critical positive role in vascular endothelial survival, health and growth. However, several of these studies were performed using cultured ECs *in vitro*, global knockdown animal models of NADPH oxidase (p47phox−/− or gp91phox−/−), or constitutive over-expression of NADPH oxidases (e.g. Nox4) in ECs[9, 10, 12, 14, 16, 35, 36]. These approaches, although yielding important information, may have precluded precise determination of endothelial contribution for redox-sensitive regulation of vascular functions.

To examine endothelial responses to conditional *in vivo* increase in EC-specific endogenous ROS in adult animals, we have generated a novel binary (Tet-ON/OFF) transgenic mouse that induces EC-specific 1.8±0.42-fold increase in Nox2/gp91phox (NADPH oxidase 2)-derived ROS for four to 12 weeks. Using isolated mouse heart EC (MHEC) and coronary microvessels, we demonstrate that conditional increase in EC-specific ROS induces AMPK-eNOS-mediated endothelium-dependent coronary vasodilatation, and AMPK-mTOR-mediated protective autophagy in MHEC.

## RESULTS

### Nox2 overexpression in the vascular endothelium

**Figure 1:**
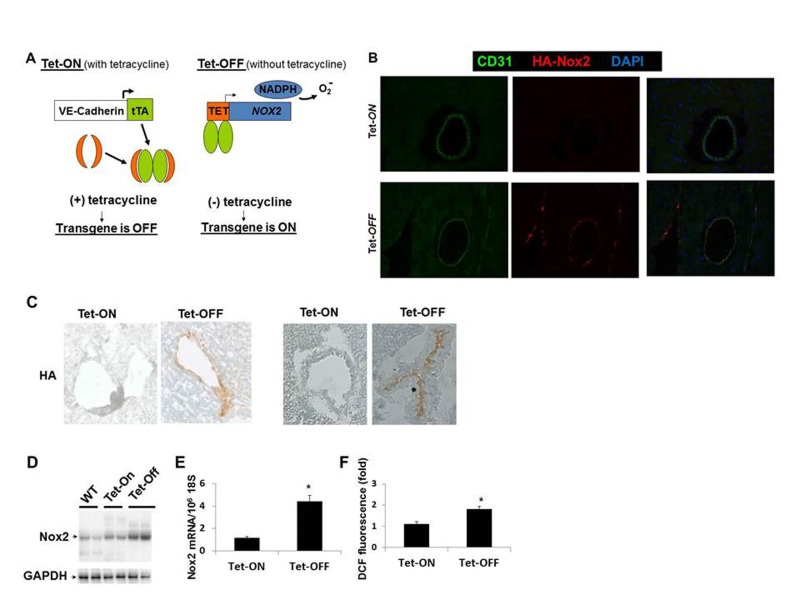
Endothelium-specific overexpression of Nox2 and ROS generation **(A)** Schematic used to make conditional binary transgenic mice (NVF). Tet-ON, tetracycline in the drinking water to suppress the transgene; Tet-OFF, withdrawal of tetracycline for four weeks to induce the transgene. **(B)** Frozen heart sections of 8 weeks old Tet-ON and Tet.OFF NVF (for 4 weeks) mice showing EC-specific (CD31, green) HA-tagged Nox2 (red) expression in coronary vessels of Tet-OFF animal. **(C)** Immunohistochemistry using anti-HA antibody on frozen heart sections. **(D)** Western blots using transgenic mouse heart EC (MHEC) lysates showing Nox2 overexpression in two independent lines of Tet-OFF animals compared to Tet-On and WT animals. **(E)** Q-PCR using MHEC RNAs from Tet-ON and Tet-OFF animals (n=6/group). **(F)** FACS analyses using DCF fluorescence of MHECs (n=6/group). All animals were 8 weeks old, Tet-OFF were without tetracycline for four weeks. Tet-ON MHECs were grown in medium containing tetracycline 2μg/mL. *p<0.05.

The double/binary transgenic NVF (TRE-NOX2:VE-Cadherin-tTA) animals were supplied with tetracycline in the drinking water to turn off the transgene expression as described in the Materials and Methods (Fig. 1A). Withdrawal of tetracycline from the drinking water (Tet-OFF) for at least 2 weeks induced NOX2-HA specifically in the endothelium of the double transgenic mice (Fig. 1B-1C, shows Tet-OFF for 4 weeks. Supplemental Fig. 1A shows Tet-OFF for 8 weeks). In order to examine the effects of Nox2 overexpression in ECs, mouse heart endothelial cells (MHEC) were isolated from two independent binary transgenic mouse lines as described [10]. MHECs from Tet-ON animals were grown in medium containing tetracycline (2 μg/ml) and Tet-OFF MHEC were grown in medium without tetracycline. Western blots using protein lysates and RT-PCR using RNA from Tet-OFF MHEC demonstrated significantly increased levels of Nox2 protein and Nox2 mRNA, respectively, compared to Tet-ON MHEC (Fig. 1D-1E). To determine whether EC-specific Nox2 overexpression increased ROS levels, DCF-DA fluorescence assays using FACS were performed. Tet-OFF MHEC showed 1.8±0.42-fold increase in ROS levels compared to Tet-ON MHEC (Fig. 1F), showing that overexpression of Nox2 resulted in increased ROS levels in ECs. However, there were no significant changes in the expression levels of other components of NADPH oxidase complex tested such p47^phox^, p22^phox^ or nox4 (data not shown). Si-RNA knockdown of Nox2 in Tet-OFF MHEC resulted in significant inhibition of NADPH oxidase activity (Supplemental Fig. 1B) and decrease in ROS levels (Supplemental Fig. 1C), suggesting that increased ROS levels observed in Tet-OFF MHEC were due to increase in Nox2-mediated NADPH oxidase activity. In order to confirm specificity of DCF-fluorescence by ROS, catalase but not L-NAME was shown to inhibit ROS in Tet-OFF MHEC (Supplemental Fig. 1D). Together, these data demonstrate that the binary transgenic mouse (NVF) can generate increased levels of endogenous ROS in ECs upon withdrawal of tetracycline.

### Increased coronary vasorelaxation in Tet-OFF mice

**Figure 2:**
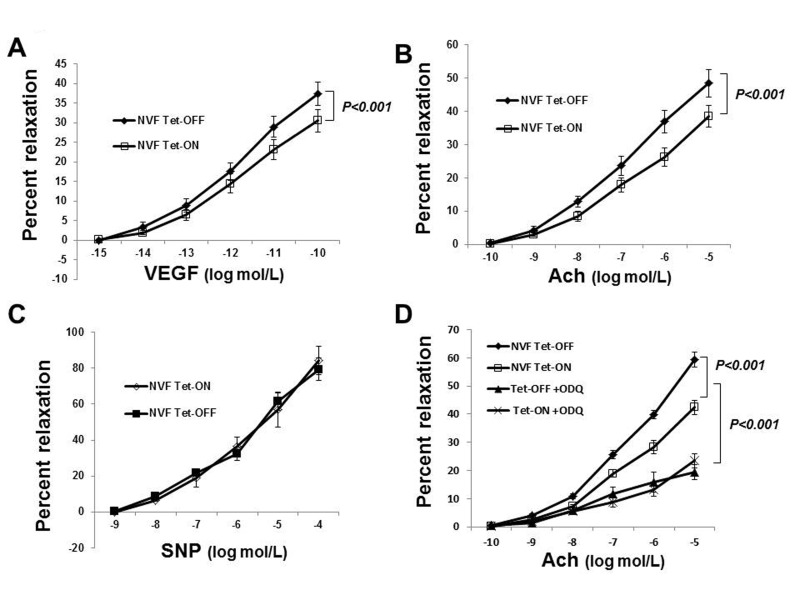
Increased coronary vasodilatation in Tet-OFF mice with higher EC-ROS **(A)** Endothelium-dependent dilation of coronary arterioles from Tet-ON (n=6) and Tet-OFF (n=6) NVF mice in response to VEGF. 22±3.72% increase in vasodilation in Tet-OFF vs. Tet-ON. **(B)** Endothelium-dependent dilation of coronary arterioles from Tet-ON (n=6) and Tet-OFF (n=6) NVF mice in response to acetylcholine (Ach). 25±2.43% increased dilation in Tet-OFF vs. Tet-ON. **(C)** Endothelium-independent dilation of coronary arterioles from Tet-ON (n=6) and Tet-OFF (n=6) mice in response to NO donor, SNP. **(D)** NO-cGMP signaling inhibitor ODQ (10 μmol/L) inhibited coronary vasorelaxation in both Tet-ON and Tet-OFF coronary vessels. n = 6/group. All coronary vessels were pre-constricted *ex-vivo* using U46619 prior to the addition of VEGF, Ach or SNP as indicated.

The established dogma or classical belief is that ROS reduce bioavailability of NO by converting it to peroxynitrite (ONOO) and thus inhibit endothelium-dependent vasorelaxation. There are, however, conflicting reports about the roles of ROS in vascular biology. Recent findings by others [37] and us [10] demonstrated that NADPH oxidase-derived ROS are essential for endothelium-dependent vasorelaxation. Failure of the clinical trials using antioxidants to improave the health of cardiovascular patients also suggested a critical role for ROS in vascular function [5-8]. To determine whether EC-ROS resulted in the improvement of coronary vasorelaxation or not, we isolated coronary microvessels from Tet-ON and Tet-OFF NVF transgenic mice (8-12 weeks old) and subject endothelium-dependent microvessel reactivity assays using vascular endothelial growth factor (VEGF) and acetylcholine (Ach) as described [10]. Tet-OFF coronary vessels showed significantly increased (by >22%) vasodilation over Tet-ON vessels in response to VEGF and Ach, however, there was no difference in SNP induced relaxation in these vessels (Fig. 2), suggesting that increased ROS levels in EC resulted in endothelium-dependent vasodilatation in coronary vessels. Non-specific effects of tetracycline on Tet-ON animals were excluded by performing microvessel reactivity assays on coronary vessels from WT animals that were treated with or without tetracycline for eight weeks (Supplemental Fig. 2A). Endothelium denudation and eNOS-inhibitor L-NAME (300 μmol/L) inhibited microvessel reactivity in both Tet-ON and Tet-OFF coronary vessels (data not shown), suggesting endothelium-generated NO plays a major role in coronary vasorelaxation. Inhibition of vasodilatation in Tet-OFF animals by another NO-scavenger carboxy-PTIO (200 μmol/L) (Supplemental Fig. 2B), further supported this notion. Supplementation of drinking water with tetracycline in Tet-OFF mice for 4 weeks (Tet-OFF+Tet-ON) and incubation of coronary vessels with ROS-scavenger NAC reduced vasodilation in Tet-OFF animals to the level of that of Tet-ON mice (Supplemental Fig. 2B), suggesting specific role for NADPH oxidase-derived inducible ROS in vasodilatation in these binary transgenic animals. One may wonder whether increased level of ROS and NO resulted in higher ONOO^−^ levels in Tet-OFF vessels, which in turn was responsible for increased vasorelaxation. To address this, microvessel reactivity assays were performed using nitric oxide-cGMP inhibitor, ODQ (10 μmol/L). ODQ inhibited vasodilatation in both Tet-ON and Tet-OFF coronary arterioles (Fig. 2D). Together with c-PTIO data (Supplemental Fig. 2B), these findings suggest that NO, but not ONOO^−^, plays a major role in EC-ROS-mediated increase in vasorelaxation.

### Sustained increase in EC-ROS induces eNOS activation by AMPK

**Figure 3:**
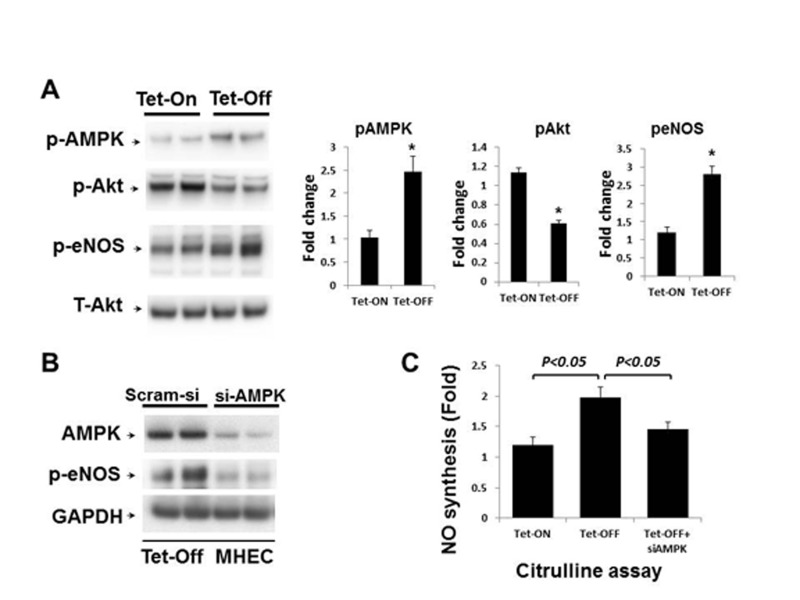
Above-physiological ROS levels induce AMPK-mediated eNOS activation in Tet-OFF MHEC (**A**) Western blots (WB) analyses of MHEC protein lysates from two independent lines of NVF Tet-ON and Tet-OFF mice as indicated. WB was carried out using anti-phospho-AMPK (p-AMPK), anti-p-Akt (ser473), anti-p-eNOS (ser1179) and anti-T-Akt (total) antibodies. T-Akt was used as loading control. Right panels, bar graphs show quantitative densitometric analysis of three independent experiments using NIH image J (-fold change expressed in mean ± S.E.M.). *p<0.05 was considered statistically significant. (**B**) Protein extracts from Tet-OFF MHEC transfected with control siRNA (Scram-si) or si-AMPK were subject to Western blots as described in the Methods. Membranes were sequentially blotted, stripped and re-probed with anti-AMPK, anti-p-eNOS and GAPDH antibodies as shown. Representative blots of two independent experiments are shown. **(C)** NO production, as measured using citruline assay as described in Methods, was 2.1±0.32-fold higher in Tet-OFF MHEC compared to Tet-ON. Si-AMPK significantly inhibited NO production in Tet-OFF MHEC. *p<0.05.

In order to examine the effects of increased ROS on eNOS activation and NO bioavailability, Tet-ON and Tet-OFF MHEC lysates were subject to Western blot analysis. Tet-OFF MHEC demonstrated increased phosphorylation of eNOS (Ser1179) in an AMPK-dependent manner (Fig. 3A-3B). However, there was no difference in total eNOS expression between Tet-ON and Tet-OFF MHEC (Supplemental Fig. 3). AMPK-dependent eNOS activation by EC-ROS was further confirmed by demonstrating increased NO levels in Tet-OFF MHEC and its inhibition by si-AMPK, as determined by citrulline assay (Fig. 3C). Akt phospho-rylation remained unchanged (Fig. 3A). Together, these fidnings suggest increase in EC-ROS results in activation of eNOS in an AMPK-dependent mechanism.

### Increased coronary vasorelaxation in Tet-OFF mice is AMPK-dependent

**Figure 4:**
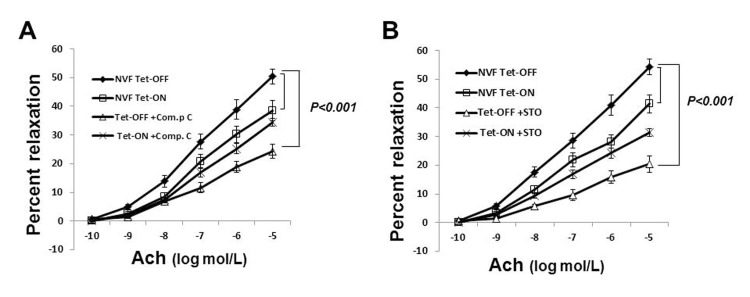
Inhibition of AMPK signaling reduced coronary vasodilatation in Tet-OFF NVF but not in Tet-ON NVF **(A)** Isolated coronary vessels from Tet-ON and Tet-OFF transgenic mice (n=6/group) were subject to microvessel reactivity assay in the presence or absence of Compound C (80 μmol/L). Ach-mediated vasodilatation was inhibited by Compound C in the coronary vessels from Tet-OFF mice, whereas Compound C had no significant effect on the coronary vessels from Tet-ON mice. **(B)** Same as in (A), except pre-treatment was carried out using CaMKKβ-inhibitor STO-609 (50 nmol/L).

Since eNOS activation in Tet-OFF MHEC is AMPK-dependent, we wanted to examine whether the increase in coronary vasorelaxation in Tet-OFF NVF mice was AMPK-mediated. To that end, isolated coronary vessels from Tet-ON and Tet-OFF transgenic mice were subject to microvessel reactivity assay in the presence or absence of AMPK inhibitor, Compound C (80 μmol/L). Ach- and VEGF-mediated coronary vasodilatation was inhibited by Compound C in Tet-OFF mice, whereas Compound C had no significant effect on the coronary vessels from Tet-ON mice (Fig.4A shows Ach-mediated microvessel reactivity). These results suggest that the observed increase in Tet-OFF coronary vasodilatation was, at least in part, induced by an AMPK-mediated endothelium-dependent signaling pathway.

### ROS-induced AMPK-eNOS Activation is CaMKK*β*-dependent

Earlier studies demonstrated that CaMKK acts as an upstream kinase for AMPK in ECs. Since ROS-stimulated eNOS activation was AMPK-dependent, we hypothesized that CaMKKβ was likely to be the upstream kinase involved in AMPK activation. To examine selective role of CaMKKβ in ROS-induced AMPK activation, CaMKKβ was knocked down by si-CaMKKβ. Transfection with si-CaMKKβ significantly reduced AMPK Thr-172 phosphorylation in Tet-OFF MHEC (Supplemental Fig. 4), suggesting that AMPK activation by ROS is CaMKKβ-dependent. STO-609 (50 nmol/L) is a potent inhibitor of CaMKK (both CaMKKα and CaMKKβ). This low concentration of STO-609 was shown to be selective for CaMKK, and did not inhibit other kinases including AMPK. STO-609 inhibited endothelium-dependent vasorelaxation in Tet-OFF mouse coronary arterioles (Fig. 4B), suggesting that ROS-induced AMPK-eNOS-mediated vasodilatation is CaMKK-dependent.

### Increased EC-ROS inhibit mTOR-p70S6K signaling

**Figure 5:**
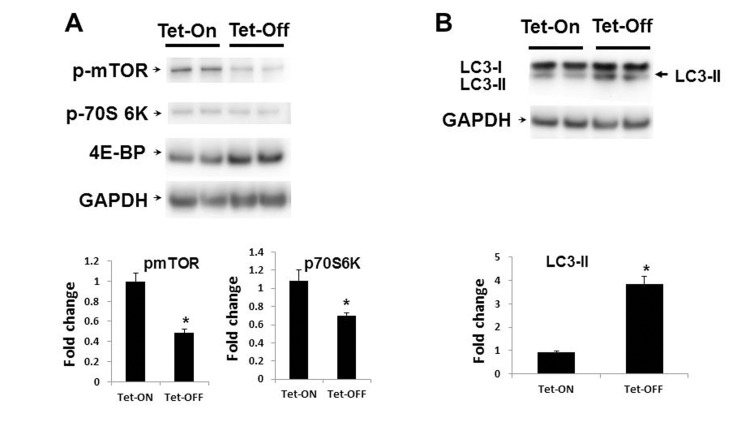
Inhibition of mTOR signaling in Tet-OFF MHEC with high ROS (**A**) Western blot analyses of MHEC protein lysates from two independent lines of NVF Tet-ON and Tet-OFF mice as indicated. WB was carried out using anti-p-mTOR (p-mTOR), anti-p-70S 6k, and anti-4E-BP antibodies. GAPDH was used for loading control. Lower panels, bar graphs show quantitative densitometric analysis of three independent experiments of the p-mTOR and p-70S 6K bands (-fold change expressed in mean ± S.E.M.). *p<0.05 was considered statistically significant. (**B**) WB analyses of MHEC from two independent lines of NVF Tet-ON and Tet-OFF as in (A) except anti-LC3A (I and II) and ant-GAPDH antibodies were used. Arrow, induction of the autophagy marker LC3-II in Tet-OFF MHEC is indicated. Lower panel, bar graph showing quantitative analyses of LC3-II as indicated. *p<0.05.

AMPK activation is known to have inhibitory effects on mTOR signaling[38, 39]. Since Tet-OFF MHEC with higher levels of ROS demonstrated increased activation of AMPK, we examined whether downstream mTOR/p70S6K/eIF-4E-BP signaling were also affected. Western blots analysis demonstrated marked reduction in mTOR/p70S6K signaling in MHEC with higher ROS (Fig. 5A). Reduction in mTOR signaling was further confirmed by increased levels of 4E-BP, a protein which is degraded by mTOR-mediated phosphorylation, in Tet-OFF MHEC (Fig. 5A). Together, these results suggest that higher levels of NADPH oxidase-derived ROS inhibit mTOR signaling pathway in ECs.

### Increased ROS induce autophagy in EC

**Figure 6:**
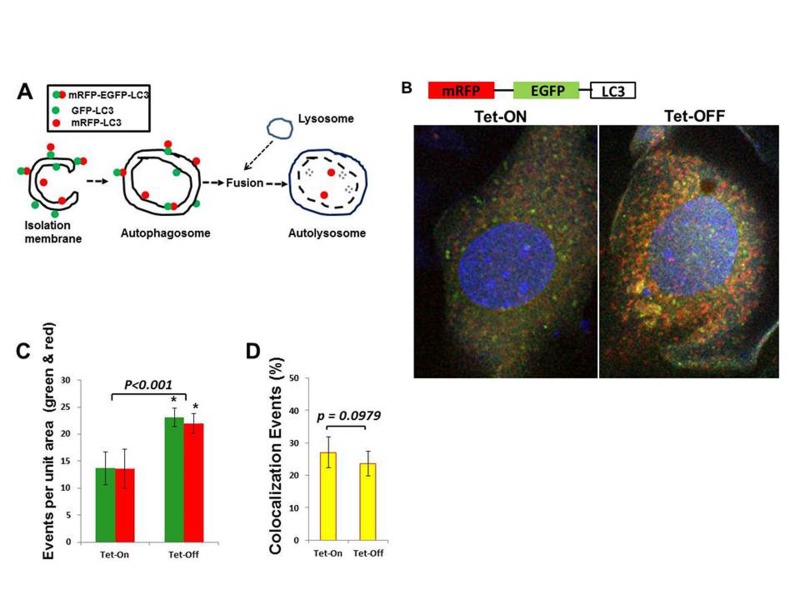
Increased autophagy in Tet-OFF MHEC using adenovirus expressing mRFP-GFP-LC3 **(A)** Schematic presentation shows that GFP-LC3 (green) and mRFP-LC3 (red) signals are present on the autophagosome, whereas autolysosome contains only mRFP-LC3 (red) signals [40]. **(B)** Confocal microscopy of Adv-mRFP-GFP-LC3-transduced MHECs. **(C)** Quantification of red and green fluorophores using NIH ImageJ 1.47b demonstrate >1.5-fold increase in autophagy in Tet-OFF MHEC (n=50 cells). **(D)** Quantification of colocalization events (yellow) using spatial overlap of red and green was done in Fiji program (ImageJ 1.47h). 1.5-fold increase in overall autophagy in Tet-OFF MHEC (C), but no significant changes in the ratio of intracellular *red vs. green* in Tet-OFF MHEC (C), and no changes in the colocalization signals between *Tet-ON vs. Tet-OFF* (D) suggest an effective autophagic flux in Tet-OFF MHEC. *p<0.05.

Since MHEC with higher ROS levels showed activation of AMPK and inhibition of mTOR signaling pathways, we hypothesized that Tet-OFF MHEC would demonstrate increased autophagy. Western blots showed a significant increase in the autophagosome-bound LC3A-II in Tet-OFF MHEC compared to Tet-ON MHEC (Fig. 5B), suggesting an increase in autophagosome formation in EC with higher ROS. To further confirm this finding, we transduced MHEC with a construct containing mRFP-EGFP-LC3 that gives off green and red fluorescence signals upon autophagosome formation, but gives off only red signal when autophagosome becomes fused with lysosome to form autolysosome (acidic pH in autolysosome quenches green signal) [25, 40] (Fig. 6A). In accordance with suppression of mTOR signal and increase in LC3A-II, Tet-OFF MHEC demonstrated significant increase in the autophagic events as determined by increased green/red fluorescence compared to Tet-ON MHEC confocal microscopy (Fig. 6B). Quantification of red and green fluorophores showed 1.5±0.18-fold increase in overall autophagy (red plus green signals) in Tet-OFF MHEC compared to Tet-ON MHEC (Fig. 6C). There were, however, no significant changes in the intracellular ratio of red *versus* green signals in Tet-ON MHEC or in Tet-OFF MHECs (Fig. 6C), and the percentages of colocalization events (overlapping green and red signals) between Tet-ON and Tet-OFF MHEC were similar (Fig. 6D), suggesting that increased autophagy in Tet-OFF MHEC was accompanied by increased autophagosome and autolysosome formation. Together, these data suggest an increased autophagic flux in Tet-OFF MHEC.

### ROS-induced autophagy plays a protective role in EC survival

**Figure 7:**
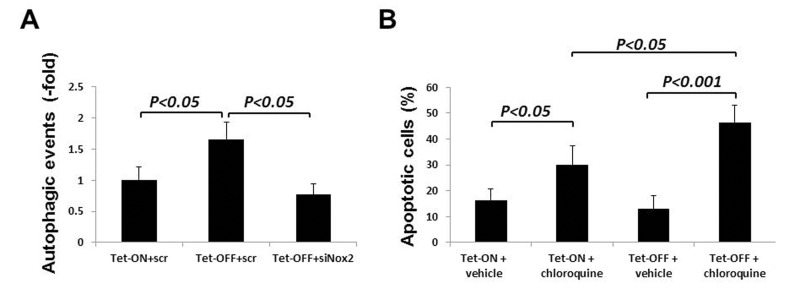
Nox2-induced autophagy plays a protective role in cell survival during oxidative stress in ECs **(A)** Tet-ON and Tet-OFF MHEC were transduced with Adv-mRFP-GFP-LC3 and transfected with either si-scr or si-Nox2 as indicated. Quantification of red and green fluorophores using NIH ImageJ 1.47b demonstrate significant increase in autophagy in Tet-OFF MHEC (n=50 cells), which was abrogated by knockdown of Nox2. **(B)** Annexin V-FITC labeling was carried out to determine apoptotic MHEC as described in the Methods. Bar graph shows apoptotic cells as percentage of total viable cells population using three independent experiments. Autophagosome-lysosome fusion blocker chloroquine induced apoptosis in both Tet-ON and Tet-OFF MHEC. However, chloroquine-induced apoptosis was significantly higher in Tet-OFF MHEC.

To determine whether increased autophagy was due to increased ROS levels, we transfected Tet-OFF MHEC with siRNA-Nox2 (si-Nox2) or pre-treated with NAC (300 μmol/L). Reduction in ROS resulted in inhibition of autophagy in Tet-OFF MHEC (Fig. 7A shows data using si-Nox2), suggesting a role for Nox2-derived ROS in increased autophagy in Tet-OFF MHEC. Next, we wanted to examine whether increased autophagy played a protective for the survival of MHEC with higher ROS levels. Tet-OFF MHEC were treated with chloroquine to inhibit autolysosome formation (chloroquine prevents acidification of lysosomes resulting in inhibition of proteolytic activitiy). Chloroquine-treated Tet-OFF MHEC had significantly higher levels of apoptotic cell death compared to vehicle-treated Tet-OFF MHEC (Fig. 7B), suggesting a protective role for increased autophagy in the survival of ECs with high ROS levels.

## DISCUSSION

Here, we demonstrate that above-physiological levels of NADPH oxidase-derived ROS in vascular endothelium *in vivo* induce activation of the pro-survival kinase AMPK, activation of downstream eNOS, and endothelium-dependent coronary vasodilatation. We specifically show that downstream of AMPK, eNOS activation and mTOR inhibition have two distinct positive effects on vascular endothelium during oxidant stress: (i) redox-AMPK-eNOS signaling increases nitric oxide levels to improve endothelium-dependent vasodilatory response in coronary vessels; and (ii) redox-AMPK-mTOR signaling appears to play critical role in endothelial survival by inducing protective autophagy.

These results demonstrate that increased ROS levels improve vascular function. This contradicts the currently believed ‘ROS theory’ and argues against oxidative damage as a probable initial cause of age-associated microvascular pathology. Several recent elegant studies have also challenged the ‘ROS theory’ of aging and organ damage [41-43]. The data presented in this study are obtained using conditional increase (1.8-fold) in NAPDH oxidase-derived ROS in a vascular endothelium-specific manner for four to 12 weeks. There were no changes observed in gross phenotype, fertility or blood pressure in these animals.

The findings suggest existence of an endothelial compensatory mechanism(s) that prevents oxidative damage to the vasculature. Increased synthesis of NO and autophagic flux, as reported in this study, appear to play critical roles in resetting endothelial signaling pathways during oxidative stress. However, further studies are required to determine the effects of prolonged exposure of vascular ECs (beyond 12 weeks) to above-physiological ROS levels that result in ‘hyperfunction’ [41] of AMPK-eNOS (i.e. NO synthesis) and AMPK-mTOR (i.e. autophagy).

Using *in vitro* cell culture, w*e* and others have previously reported that NADPH oxidase-derived ROS in ECs are involved in the activation of several signaling pathways including PI3K-Akt-eNOS [9, 10, 12, 26, 44]. In the current study, we did not find significant alteration in Akt phosphorylation in the mouse heart ECs (MHEC) with higher redox content, suggesting an apparent disconnect between *in vitro* and *in vivo* systems. Interestingly, increased endothelial ROS levels resulted in the activation of the survival kinase AMPK and downstream eNOS. Activation of eNOS by AMPK has been reported earlier in several cardiovascular cells including ECs [45, 46]. AMPK, a kinase known to ‘sense’ cellular metabolic state (AMP *vs.* ATP), is activated in several cell types following caloric restriction, hypoxia/ischemia, heat shock, and exercise [47, 48]. Two upstream major kinases, LKB1 and CaMKKβ, have been shown to activate AMPK. However, AMPK's role in ECs as a metabolic sensor has not yet been shown and the precise role of AMPK in EC remains incompletely understood. CaMKKβ-mediated activation of AMPK has been shown to occur independent of AMP levels in ECs[49]. Our data support CaMKKβ-mediated AMPK activation in EC with high redox content. To our knowledge, this is the first study demonstrating AMPK activation in ECs in response to increased levels of NADPH oxidase-derived endogenous ROS.

The role of AMPK in the maintenance of vascular tone and vasodilator function remains controversial. Global knockout of AMPK did not affect NO-mediated arterial vasorelaxation in mice[50]. On the contrary, AMPK activator metformin was shown to improve vasodilatation[51, 52]. In addition, several compounds including fenofibrate, rosiglitazone, cilostazol, that activate AMPK were also shown to improve EC-dependent vasodilatation. Resveratrol, a polyphenol known for its cardio-protective and positive survival effects[53], has been reported to increase AMPK activity. The precise mechanism by which AMPK activation brings about these positive changes in vascular health is not known. In the present study, using AMPK-inhibitor compound C and cGMP-inhibitor ODQ, we have shown that ROS-mediated, endothelium-dependent vasodilatation in coronary vessels occurs through activation of AMPK-eNOS-NO axis. These findings suggest an important role for AMPK in the regulation of coronary vasorelaxation during oxidative stress in the vascular endothelium.

Caloric restriction or nutrition deprivation slows down energy-consuming processes and induces autophagy to provide amino acids for the synthesis of essential proteins, resulting in cell survival and inhibition of apoptosis under stressful conditions such as oxidative stress in many cell types. Autophagy is essential for cellular survival, homeostasis, differentiation, and tissue remodeling in physiological and also in pathological conditions. However, depending on the patho-physiological settings, autophagy may play a protective role or contribute to cell damage. Our novel findings that increased ROS levels elicit a ‘caloric restriction’-like response (AMPK-mediated mTOR inhibition and induction of autophagy) in endothelium may have important clinical implication. It is plausible that endothelium deals with higher redox state by slowing down endothelial metabolism and/or by recycling the oxidant-damaged cellular organelles. Further studies are required to address this issue.

**Figure 8:**
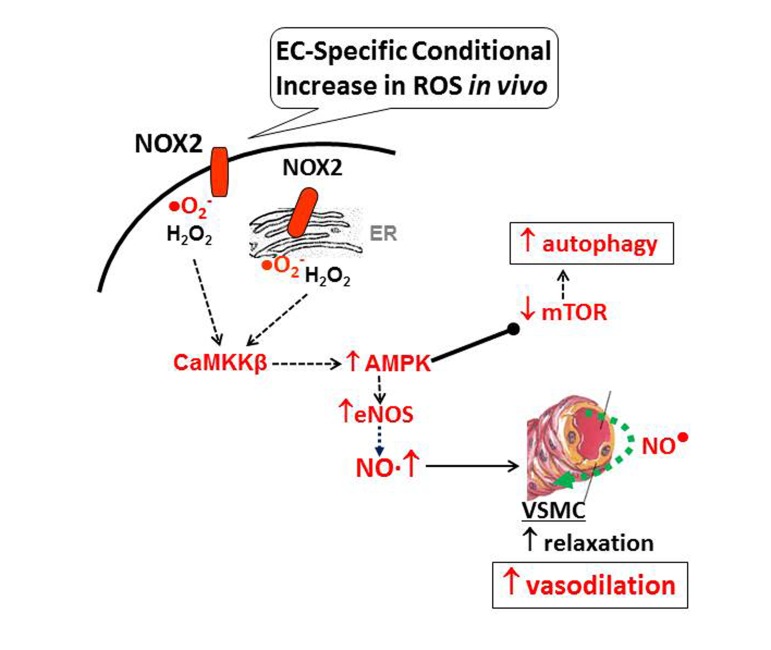
Model for EC-specific ROS-mediated improvement in endothelial function Nox2-induced ROS in vascular endothelium activates *CaMKK*β-AMPK, which in turn, activates eNOS to induce NO-mediated vasodilatation and inhibits mTOR resulting in protective autophagy.

Taken together, the findings reported here suggest that NADPH oxidase-derived increase in endothelium-specific ROS induces AMPK-eNOS signaling, NO synthesis and coronary vasodilatation (Fig. 8). Increased ROS also elicit protective mechanisms in coronary endothelium that include AMPK-mTOR-induced autophagy. These findings raise several important questions that need to be addressed in future: (a) what effects would sustained increase in endothelial ROS (beyond 12 weeks) have on coronary vascular function? (b) Do EC-ROS have similar effects on other vascular beds? Preliminary data using aorta demonstrate similar vasodilatory response in Tet-OFF animals (Supplemental Fig. 5). (c) What effects do increased ROS levels have on AMPK-regulated signaling intermediates and transcription factors in vascular endothelium, namely PGC-1α, HIF-1α, and FOXO1? (d) Will redox-AMPK axis modulate endothelial cell growth and proliferation, and thus angiogenesis, in ischemic tissues that often have higher ROS levels, such as IHD? Ongoing studies in our lab are addressing these important questions.

## METHODS

### Generation of binary Tet-NOX2: VE-Cadherin-tTA mice

All animal experiments were approved by the Lifespan Institutional Animal Care and Use Committee. The binary tetracycline (Tet)-ON/Tet-OFF system and VE-Cadherin promoter were used to render the transgene inducible and endothelium-specific, respectively [54]. In brief, the HA-tagged NOX2 gene was placed downstream of a Tet (TRE) promoter in one mouse (Tet-NOX2) and the tTA gene was placed under a VE-Cadherin promoter in another mouse (VE-Cad-tTA) (Fig. 1A). Cross-breeding of Tet-Nox2 and VE-Cad-tTA mice were carried out to obtain double transgenic NVF (Tet-Nox2:VE-Cad-tTA) mice. To identify double-positive NVF transgenic mice, PCR was carried out using Platinum^®^ PCR Supermix (Invitrogen). The primer sequences flanking Tet-responsive element (TRE) upstream and Nox2 ORF downstream were used: for sense primer 5'- AGA GAA AAG TGA AAG TCG AGC TCG GT -3', and for antisense primer 5'- TGT GCA GTG CTA TCA TCC AAG C -3'. The primer sequences for VE-Cadherin-tTA were: Sense – 5'- CAG TAG TAG GTG TTT CCC TTT CTT -3', and antisense – 5'- GAC GCC TTA GCC ATT GAG AT -3'. Expression of the transgene (NOX2) was turned-off in double transgenic (NVF) by adding tetracycline (Tet-ON) in the drinking water since *in utero*. Withdrawal of tetracycline (Tet-OFF) for at least two weeks was required to achieve ~4-fold increase in expression level of Nox2 mRNA and ~1.8-fold increase in ROS levels in ECs (data not shown). For all experiments reported here, tetracycline (Tet) was withdrawn (Tet-OFF) for a minimal period of two weeks to a maximum period of 12 weeks, whereas the sex-matched littermates on Tet were used as control (Tet-ON). HA-tag was used to differentiate between the endogenous gene product and the transgene. All experiments were carried out using age- and sex-matched (male) animals. Two independent lines of the NVF transgenic mice were used in the current study.

### Immunofluorescence and immunohistochemistry assays

Immunofluoresence and immunohistochemistry assays were performed as described [55, 56]. Briefly, tissues were harvested and embedded in HistoPrep^TM^ frozen tissue embedding media. Frozen sections were immunostained using anti-CD31 antibody (BD Pharmingen^TM^, 1:250) followed by anti-Rat Alexa 488 secondary antibody. Anti-HA antibody was used to detect expression of the transgene (Novus Biologicals, 1:3000), Microscopy was performed and the Spot Advanced Software was used to capture images in red, green and blue channels. Composite images were also created using Spot Advanced.

### Ex Vivo coronary microvessel relaxation studies

After cardiac harvest from 8- to 12-week-old Tet-ON (control) and Tet-OFF Tet-Nox2:VE-Cad-tTA (NVF) mice, coronary arterioles (diameter, 80 to 120 μM; length, 2 mM) were dissected from the surrounding tissue. Microvessel studies [6 mice from each group (Tet-ON and Tet-OFF)] were performed using *ex vivo* organ bath videomicroscopy, as previously described [10]. Vessels were precontracted using U46619. Where indicated, isolated coronary vessels were pretreated with NO-inhibitor L-NAME (300 μmol/L), ROS inhibitor NAC (400 μmol/L), NO-cGMP inhibitor ODQ (10 μmol/L) and AMPK inhibitor Compound C (80 μmol/L). Endothelial denudation was carried out by advancing a human hair into the lumen of the vessel and gently abrading the luminal surface.

### Mouse heart EC isolation and culture

Mouse heart ECs (MHECs) were isolated from the heart specimens of Tet-ON and Tet-OFF animals, as previously described [10]. For each experiment, primary cultures of Tet-ON and Tet-OFF were started simultaneously (a pool of 3 hearts from each group). Tet-ON MHEC were grown in DMEM-based EC culture medium containing tetracycline (2 μg/mL). Results were obtained, in triplicate, using three independent batches of MHEC isolation per group (Tet-ON and Tet-OFF).

### Autophagosome/autolysosome formation assay

Tet-ON and Tet-OFF MHEC grown on Lab-Tek Chamber slides were transduced with Ad- mRFP-EGFP-LC3 at 5 MOI (multiplicity of infection) [25, 40]. After 36 hours, MHEC were washed twice with PBS and fixed with 4% paraformaldehyde. DAPI containing mounting medium (Vectashield) was used to identify nuclei. Fluorescence and confocal micrsocopy were used to capture images from three different MHEC preparations for quantification of green, red, and overlapped green-red signals. NIH ImageJ 1.47b was used for quantification of red and green fluorophores (n=50 cells). Fiji program (ImageJ 1.47h) was used to quantify colocalization events (yellow) using spatial overlap of red and green.

### Western blots

Cell lysates were prepared from MHEC grown on 0.1% gelatin-coated plates to 80%-90% confluence. Western blot (WB) analyses using MHEC protein lysates were performed as previously described [9, 10]. Anti-phosphorylated (phos) (S473) Akt, anti-phos (S1179) eNOS, anti-phos (T172) AMPK, anti-phos (S2481) mTOR, anti-phos (T389) P70S6K, anti-Nox2, anti-Akt, anti-4E-BP, and anti-LC3A, anti-GAPDH and anti-AMPK antibodies were obtained from Cell Signaling (Beverly, Mass), Abcam (Cambridge, Mass), and Thermoscientific (West Palm Beach, FL). WBs were performed using lysates from three independent experiments and were analyzed using NIH Image J for quantification. * *p* < 0.05. Representative blots are shown in the Results section.

### NADPH oxidase activity and DCF fluorescence assays

Tet-ON and Tet-OFF MHEC were washed with ice-cold PBS, collected by a cell scraper, and Dounce-homogenized in buffer containing 20 mmol/L KH_2_PO_4_ (pH 7.0), 1× protease mixture inhibitor (Sigma), 1 mM EGTA, 10 μg/mL aprotinin (Calbiochem), 0.5 μg/mL leupeptin (Sigma), 0.7 μg/mL pepstatin (Sigma), and 0.5 mmol/L phenyl-methylsulfonyl fluoride (Calbiochem). NADPH oxidase activity was measured using a modified assay [57]. Briefly, photon emission from the chromogenic substrate lucigenin (5 μmol/L) as a function of acceptance of electron generated by the NADPH oxidase complex was measured every 15 s for 20 min in a Berthold luminometer as described [9]. Lucigenin activity (light units/min/mg of protein) of control cells (Scram-si-transfected) was arbitrarily set at 100%. Total intracellular levels of ROS were determined by fluorescence-activated cell-sorting (FACS) analyses of the oxidative conversion of cell-permeable 2',7'-dichlorofluorescein diacetate (DCFH-DA; Molecular Probes Inc., Eugene, OR) to fluorescent dichloro-fluorescein as described previously [9]. SOD mimetic MnTBAP (200 μmol/L) and PEG-catalase (250 U/mL) were used to confirm DCF-DA fluorescence was specific to ROS (not lipid peroxides).

#### Quantitative Real Time PCR

Real time PCR was carried out as described previously[9]. Briefly, RNA was extracted from MHEC using the RNeasy RNA extraction kit (Qiagen, Valencia, CA). Total RNA (100 ng) from each of triplicate samples was converted into cDNA using random primers and SuperscriptIII reverse transcriptase (Invitrogen). Primers were designed using the Primer Express oligo design software (Applied BioSystems, Foster City, CA) and synthesized by Integrated DNA Technologies (Coralville, IA). The level of target gene expression was normalized against the 18 S rRNA expression in each sample, and the data were presented as mRNA copies per 10^6^18 S rRNA copies as described [9].

#### Cell survival assay

Apoptosis was assayed by FACS analysis of FITC-labeled annexin-V staining of Tet-ON and Tet-OFF MHEC treated with or without chloroquine (100 μmol/L) for 8 hours as described [25, 58].

### eNOS activity assay

Tet-ON and Tet-OFF MHEC were lysed in ice-cold homogenization buffer (25-mmol/L Tris·Cl, 1-mmol/L EDTA, and 1-mmol/L EGTA, pH 7.4). eNOS activity was determined using the NOS assay kit (CalBiochem, San Diego, CA) that measures conversion of L-[^3^H]arginine to L-[^3^H]citrulline as described previously [10].

### Statistical Analyses

All values are presented as mean ± SEM where appropriate. A value of P<0.05 between experimental groups was considered to represent a significant difference (*). Nonlinear regression modeling utilizing the extra sum-of-squares F test to compare slopes (Prism 5. Graph Pad Software) was used for vessel relaxation assays. ANOVA and Tukey's post-hoc test will be used for comparison between groups.

## SUPPLEMENTAL MATERIALS


