# Supplementary material for: Oxidative stress improves coronary endothelial function through activation of the pro-survival kinase AMPK
Source: Aging (Albany NY). 2013 Jun 23;5(7):515–30. doi: 10.18632/aging.100569 (PMC3765580; doi:10.18632/aging.100569)
Supplement: Supplementary file 1 [file aging-05-515-s001.pdf]

SUPPLEMENTAL MATERIALS

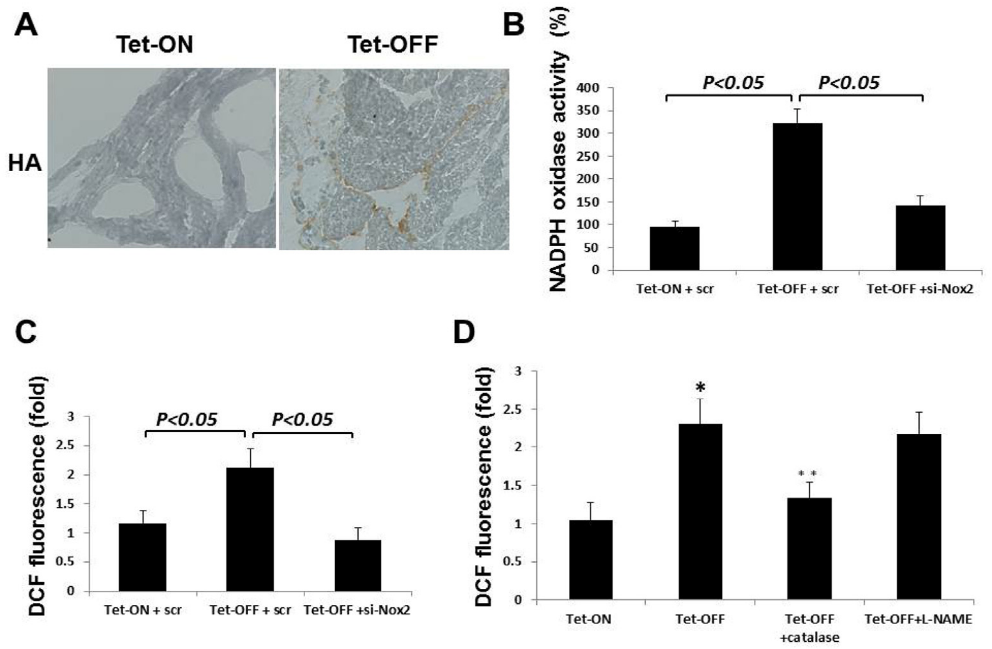

Supplemental Figure 1. NADPH oxidase activity and ROS generation in Tet-OFF MHEC is Nox2-dependent. (A) Immunohistochemistry using anti-HA antibody on frozen heart sections. Tet-OFF animals were without tetracycline for eight weeks. **(B)** MHEC were transfected with either scrambled siRNA (scr) or si-RNA against Nox2 (si-Nox2) as indicated. MHEC were subject to low-concentration (5 μmol/L) lucigenin assay to determine NADPH oxidase activity. Increased NADPH oxidase activity in Tet-OFF MHEC was significantly inhibited by si-Nox2. NADPH oxidase activity of Tet-ON MHEC was arbitrarily set at 100%. N= 3 animals/per group. **(C)** Tet-ON and Tet-OFF MHEC transfected with either scr or si-Nox2 were subject to FACS for intracellular ROS content using DCFH-DA. ROS levels of Tet-ON MHEC was arbitrarily set at 1-fold. N= 3 animals/per group. **(D)** Same as in (C), except MHEC were pre-treated with PEG-catalase (250 U/mL) and L-NAME (300 μmol/L) to confirm specificity of DCF fluorescence to superoxide/H_2O_2. * *p<0.05; **p<0.05 (*Tet-OFF vs. Tet-OFF+catalase).

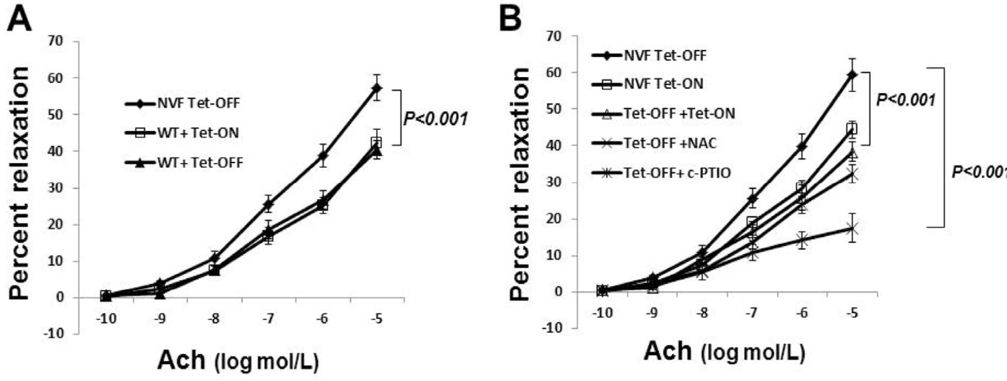

Supplemental Figure 2. Increased coronary vasodilatation in Tet-OFF mice is ROS-dependent, and requires NO. (A) Endothelium-dependent dilation of coronary arterioles from Tet-OFF (n=6) NVF mice in response to Ach is not due to non-specific effects of tetracycline. Coronary vessels from NVF Tet-OFF and from WT animals treated without (WT+ Tet-OFF) or with tetracycline (WT+ Tet-ON) for 8 weeks were subject to microvessel reactivity assays. n=6/group. **(B)** ROS scavenger NAC (400 μmol/L) reduced coronary vasorelaxation in Tet-OFF animals down to the level of Tet-ON vessels. NO scavenger c-PTIO (200 μmol/L, pH 6.9) completely inhibited coronary vasorelaxation, suggesting NO-dependence of the process. n = 6/group. All coronary vessels were pre-constricted *ex-vivo* using U46619 prior to the addition of Ach as indicated.

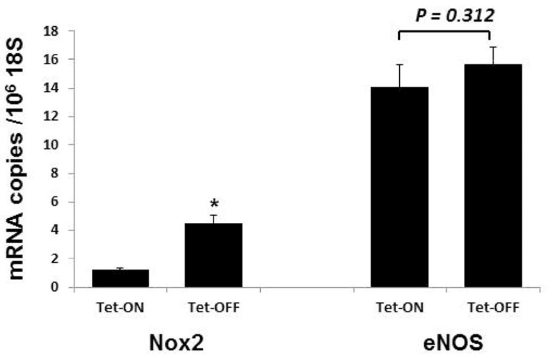

Supplemental Figure 3. Q-PCR using MHEC RNAs from Tet-ON and Tet-OFF animals (n=6/group). There was no difference in eNOS expression between Tet-ON and Tet-OFF MHECs. * p<0.05

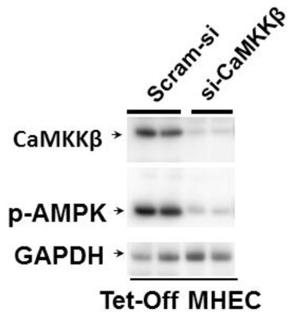

Supplemental Figure 4. AMPK activation in Tet-OFF MHEC is CaMKKβ-dependent. Protein extracts from Tet-OFF MHEC transfected with control siRNA (Scram-si) or si-CaMKKβ were subject to Western blots as described in the Methods. Membranes were sequentially blotted, stripped and re-probed with anti-CaMKKβ, anti-p-AMPK and GAPDH antibodies as shown. Representative blots of two independent experiments are shown.

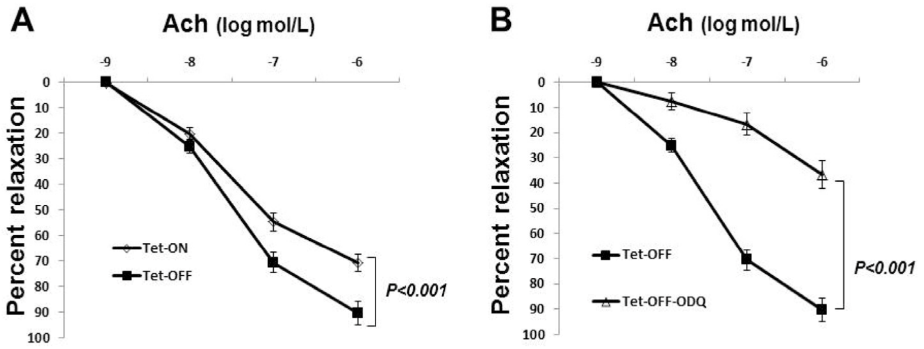

Supplemental Figure 5. Aortic ring relaxation assay using Radnoti 4 channel organ tissue perfusion bath with 4 independent isometric force transducers. (A) Preconstricted (by U46619) aortic rings from Tet-ON and Tet-OFF mice were subject to Ach as indicated and percent relaxation was analyzed using LabChart (ADInstuments). Tet-OFF aorta shows >30% increase in Ach-induced relaxation. **(B)** NO-cGMP inhibitor ODQ significantly reduces relaxation in Tet-OFF aorta, suggesting a role for NO in the process. N=4/group.
